# The influence of surface EMG-triggered multichannel electrical stimulation on sensomotoric recovery in patients with lumbar disc herniation: study protocol for a randomized controlled trial (RECO)

**DOI:** 10.1186/s13063-017-2310-z

**Published:** 2017-11-25

**Authors:** Sara Lener, Christoph Wipplinger, Sebastian Hartmann, Wolfgang N. Löscher, Sabrina Neururer, Matthias Wildauer, Claudius Thomé, Anja Tschugg

**Affiliations:** 1 Department of Neurosurgery, Innsbruck Medical University, Anichstr. 35, 6020 Innsbruck, Austria; 2 Department of Neurology, Innsbruck Medical University, Innsbruck, Austria; 3 Department of Medical Statistics and Health Economics, Innsbruck Medical University, Innsbruck, Austria; 4 Department of Neuroradiology, Innsbruck Medical University, Innsbruck, Austria

**Keywords:** Lumbar disc herniation, Sensomotoric deficit, Sensomotoric recovery, Electrical stimulation, Quantitative sensory testing, Lumbar sequesterectomy

## Abstract

**Background:**

Intervertebral disc degeneration is one of the most common reasons for chronic low back pain and sensomotoric deficits, often treated by lumbar sequestrectomy. Nevertheless, the prognostic factors relevant for time and quality of recovery, of the surgical procedure, relative to conservative treatment, remain controversial and require further investigation. Surface electrical stimulation (SES) may be an influential intervention, already showing positive impact on motor and sensory recovery in different patient groups. Since mechanisms of SES still remain unclear, further inquiry is needed.

**Methods/Design:**

This is a prospective, monocentric, randomized, controlled clinical trial. A total of 80 adult patients suffering from a lumbar disc herniation (LDH; 40 treated surgically, 40 conservatively) are allocated in a ratio of 1:1. Patients in the treatment group will receive surface electromyography (EMG)-triggered electrical stimulation for eight weeks, whereas patients in the control group will not obtain any additional treatment. The primary outcome parameter is defined as the cold detection threshold (CDT), determined by quantitative sensory testing (QST), 24 months after intervention. Secondary outcome parameters include the inquiry of sensory nerve function by two-point discrimination and QST, the assessment of motor nerve function by manual muscle testing, and validated scales and scores. These include: the Oswestry Disability Index (ODI) and the Core Outcome Measures Index (COMI) assessing the domains pain, back-specific function, work disability, and patient satisfaction; the EQ-5D investigating the patient’s generic health status; the painDETECT questionnaire (PD-Q) to identify neuropathic pain components; and the Beck Depression Inventory (BDI) to assess severity of depression. Moreover, neurological status, pain medication usage, and blood samples (CRP, TNFα, IL-1β, IL-6) will be evaluated. Study data generation (study site) and data storage, processing, and statistical analysis are clearly separated.

**Discussion:**

The results of the RECO study will detect the effect of EMG-triggered multichannel SES on the improvement of mechanical and thermal sensitivity and the effect on motor recovery and pain, associated with clinical and laboratory parameters. Furthermore, data comparing surgical and conservative treatment can be collected. This will hopefully allow treatment recommendations for patients with LDH accompanied by a sensomotoric deficit.

**Trial registration:**

ISRCTN, ISRCTN12741173. Registered on 15 January 2017.

**Electronic supplementary material:**

The online version of this article (doi:10.1186/s13063-017-2310-z) contains supplementary material, which is available to authorized users.

## Background

Intervertebral disc degeneration (IVDD) represents one of the most common reasons for low back pain (LBP), which is an increasing medical and socioeconomic problem in industrialized countries. Of the German population, 85% suffer from severe LBP at least once in their lifetime [28]. IVDD may present as lumbar disc herniation (LDH) that compresses a spinal nerve root and may lead to sensory and motor deficits. A surgical decompression of the spinal nerve root is often required in these cases. Lumbar sequestrectomy is the standard surgical procedure being performed in patients with radiculopathy caused by a prolapsed lumbar disc [31]. Nevertheless, a subsequent physical therapy to support the recovery of deficits may be needed.

A LDH, however, may regress over time with conservative treatment without the need for surgical intervention [26]. Several studies [3, 21, 37] have compared surgical to non-surgical treatment in patients with herniated discs, the SPORTS-Trial being widely quoted as a seminal article in this field of research [38]. Surgically and conservatively treated patients improved substantially over a period of 24 months. Due to a few limitations, conclusions on the superiority or equivalence of each treatment could not be drawn and further investigation is needed [38].

The patient’s sensory and motor recovery after a LDH, whether treated surgically or not, is influenced by various factors like gender, pain, duration, and severity of paresis [14, 22, 34]. Further parameters include: the degree of nerve root compression; functional recovery of nerve fibers; and potentially denervated muscle areas [27]. Electromyography (EMG)-triggered surface electrical stimulation (SES) is an intervention combining electrical biofeedback of muscles with neuromuscular electrical stimulation. It is known to be effective in improving weakness of paretic muscles and remodeling the function of the related nervous system [40]. SES is frequently used to further improve motor function in severe central and peripheral paresis, as muscle atrophy due to damage on the innervating nerve is prevented by active and passive stimulation of the muscle, as well as stimulation of the according nerve [2, 9, 19, 23, 30]. Previous studies not only showed a positive influence of electrical stimulation on promotion of peripheral nerve regeneration after injury in humans and animals [11, 15, 39], but also showed an increase in strength and muscle size in healthy subjects [20]. Additionally, patients with painful IVDD show elevated IL-6 and TNF-α levels not only in the IVDD itself [7, 17], but also in serum [36]. Electrical stimulation may lead to a downregulation of these pro-inflammatory cytokines [8, 33]. Nevertheless, the underlying mechanism of SES and its influence on sensomotoric recovery are still unclear and require further investigation.

So far, improvement of sensory and motor nerve function supported by SES has been infrequently subjected to well-designed clinical trials. Thus, there is a need for data with validated modern spinal outcome instruments (i.e. quantitative sensory testing [QST], objectively assessed muscle force, and patient’s self-assessment regarding satisfaction and ability). The objective of this prospective clinical trial is thus to evaluate the efficacy of EMG-triggered SES on sensomotoric recovery in patients with LDH, treated either conservatively or surgically, using validated modern spinal outcome instruments.

## Methods/Design

### Study design

The RECO-Study is a prospective, monocentric, randomized, controlled clinical trial. The study objective is to evaluate the improvement of sensory and motor nerve function in patients two years after either lumbar sequestrectomy (Population 1) or conservatively treated patients for LDH (Population 2), following the application of SES in the early stages of recovery (within two months after diagnosis or treatment decision). As the functional status, as well as sensory recovery parameters, are known to be assessed after two years of follow-up, this specific endpoint was also chosen for our study [12, 38, 41]. Randomization will be performed for application of electrical stimulation and therapy of recovery, but not for initial therapy of LDH. Outcome is assessed by QST [25], two-point discrimination testing (2PD) [32], manual muscle testing (MMT) [16], magnetic resonance imaging (MRI) parameters [18, 24], and by validated scales and scores. These include the Oswestry Disability Index (ODI) [6] and the Core Outcome Measures Index (COMI) [4] that are applied to assess the domains pain, back-specific function, work disability, and patient satisfaction. The generic health status is investigated with the EQ-5D [5]. Neuropathic pain components are identified with the painDETECT questionnaire (PD-Q) [10]. The severity of depression is assessed by the Beck Depression Inventory (BDI) [29]. Moreover, neurological status, muscle force, pain medication usage, and blood samples (CRP, TNFα, IL-1β, IL-6) [17, 35] will be evaluated. Furthermore, the timed up and go test (TUG) [13] will be performed. Primary and secondary outcome parameters are outlined in Additional file 1: Table S1.

### Trial organization, registration, and ethical aspects

Ethics approval was obtained from the committee of the Local Ethics Committee. This study complies with the World Medical Association Declaration of Helsinki Ethical Principles for Medical Research Involving Human Subjects, 2004, with Good Clinical Practice (GCP) and meets local laws and regulations including any and all applicable data privacy laws and regulations relating to the conduct of the study.

### Study population

The study protocol aims to include patients who suffer from a LDH with radiculopathy and a sensomotoric nerve dysfunction. Patients are naturally divided into two groups, whether their individual condition qualifies them for lumbar sequestrectomy according to the guidelines of DGNC and DGOOC (Population 1) or for initial conservative treatment (Population 2). If conservative treatment (including usage of pain medication according to the WHO level scheme and physiotherapy for all, and epidural steroid injection in exceptional cases, like therapy refractory radicular pain without motor deficit) is inefficient for more than six weeks, or if patients suffer from a severe paresis, surgical treatment will be performed. Population 2 is thus allowed to cross over to Population 1. Surgically treated patients will receive postoperative pain therapy according to the WHO level scheme but no additional active physiotherapy in the first eight weeks after surgery, except SES. Radiculopathy is defined as pain, paresis (muscle strength grade 4 or less), or a sensory dysfunction in a specific nerve root distribution area L3 to S1. Additionally, a radiologically determined pathology at treatment level investigated by MRI needs to correlate to the clinical symptoms. To minimize risk factors for an unfavorable outcome, patients with significant co-morbidities (see inclusion and exclusion criteria in, Additional file 2: Table S2) need to be excluded, as this may mask a difference in treatment efficacy. Informed consent is obtained from each participant.

### Timetable

**Fig. 1:**
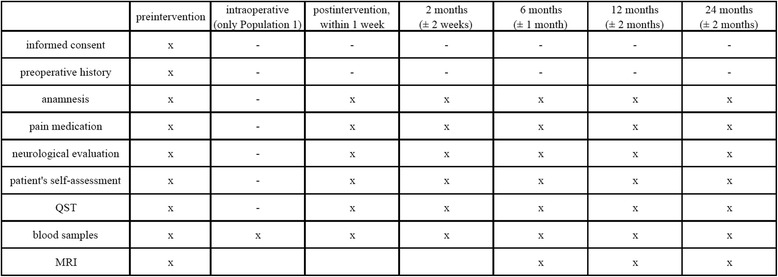
Visit plan. Detailed itemization of planned follow-ups and actions taken

Timetable and visit plan are outlined in Fig. 1.

### Surface electrical stimulation

After a guideline-dependent decision of the initial treatment for LDH, patients will be randomized into two groups, respectively, in a 1:1 allocation ratio: 20 patients (50%) in Population 1 and 20 patients (50%) in Population 2 will receive SES, whereas the other half will follow standardized treatment algorithms adjusted to the initially chosen therapy for LDH (see Additional file 3: Figure S1).

Stimulation is initially applied within three days post intervention. Patients are trained by the clinical investigator(s) at the study site to ensure proper usage of the stimulation device. Patients are then requested to perform treatment at home. Five electrodes (4 × 6.4 cm PALS latex-free neurostimulation electrodes, model 896230) are placed on the skin in the affected nerve root distribution area L3 to S1, according to instructions documented in the patient’s individual manual. For better comparability, standardized characteristic muscles for each nerve root are chosen for stimulation. M. quadriceps femoris covers distribution areas of the roots L3 and L4, M. tibialis anterior covers L5, and M. gastrocnemius is innervated by S1. Four electrodes are used to cover an as wide as possible area of each muscle, the fifth electrode is used for reference of the EMG capacity and is closely placed to bony structure (femur condyles or ankle) (see Additional file 4: Figure S2). The electrode surface of 25.6 cm^2^was chosen according to the volume of the stimulated muscles. The stimulation device (STIWELL med4, MED-EL) is started and the pre-set program (pulse width = 300 μs, frequency = 30 Hz, plateau time = 8 s, pause time = 16 s, rise time = 2 s, fall time = 2 s) is chosen. This program was developed with STIWELL specialists in the field of clinical rehabilitation and was adjusted on the condition’s special indications and expectations of recovery. The intensity will be increased until the patient feels and sees muscle contraction. If motor habituation appears, patients are instructed to increase the intensity until the comfortable tingling sensation and/or muscle contraction can be seen again. EMG triggers are imparted over the devices’ display and clearly signal the patient when to actively tense her/his muscles and when to pause. Thresholds for the EMG trigger are set at the maximum amount of electrical activity the patient is able to create. The device is set on automatic mode, which adjusts the threshold up after a successful trial or regulates it down after an unsuccessful one. Patients are instructed to perform this treatment for 1 h daily (2 × 30 min) for the following eight weeks. Time, date, and duration of each treatment should be noted in a provided document and handed out to the investigators at the next follow-up. Furthermore, treatment time, duration, and EMG thresholds can be read out directly from the device to surveil the patient’s compliance. If the treatment is conceived uncomfortable or skin irritation appears, patients are requested to immediately contact the clinical investigators.

### Sequestrectomy

Surgery is performed after induction of general endotracheal anesthesia and with the assistance of an operating microscope, while the patient is in a prone position. Surgery is performed by two trial designated surgeons in a standardized manner. The spinal canal harboring the sequestrated disc material is exposed by performing a minimal inter-laminar fenestration to avoid removal of bone and articular structures. Based on results of a previous trial, only the herniated material is removed. The herniated space is not entered whenever possible [1, 31].

### Randomization

The allocation ratio within groups is 1:1 (see Additional file 3: Figure S1). The randomization code will be generated independently from the clinical investigators according to a random permuted blocks method with varying block size. Statistical Software Stata 10.0 module Ralloc version 3.5.2 (Statacorp College Station, TX, USA) will be used to generate the random code. An independent statistician will administer the randomization code.

### Data management

Study data generation done by the clinical investigators (SL, CW, SH, WL, AT) at the study site (Department of Neurosurgery) and data storage, processing, and statistical analysis performed by the cooperating partners (SN) at the Department of Medical Statistics, Informatics and Health Economics are clearly separated. A validated database system programmed in a customized software package, which is provided by the Department of Medical Statistics, Informatics and Health Economics, is available. The evaluation of the data takes place by double-entry of the data and manual/visual evaluation of plausibility. After entry of all collected data and clarification of all queries, the database will be closed at the completion of the study.

### Sample size calculation

Sample size calculation is based on the primary endpoint of the study, the cold detection threshold (CDT) two years post intervention. Relevant data were acquired from previous prospective trials at our center, also using the CDT as a primary outcome parameter. Sixty-three patients are required to detect a difference of 3.4 considering a standard deviation (SD) of 6.8 with 80% power on a two-sided level of significance of 0.05. We considered a loss to follow-up < 20% adequate for final calculation of sample size, which is 75–80 patients.

### Statistical analyses

All statistical analyses will be performed in consultation with the Department of Medical Statistics and Health Economics, Innsbruck Medical University, Innsbruck, Austria. Detailed descriptive statistics will be provided for the data collected and 95% confidence intervals will be calculated for all relevant estimates. The primary analysis will follow as the randomized principle. All patients with a complete pre-treatment examination will be considered for inclusion into the population. Primary outcome parameter will be the CDT. We estimate that the 24 months change of the CDT in patients treated without SES will be approximately 10%, whereas patients treated with SES should show a better improvement up to 20% of the initial threshold. In a one-way analysis of variance (ANOVA) test, group sample sizes of 35 patients in each group achieve 80% power to detect a significant difference between the CDT estimates with an estimated standard deviation of 20%. To adjust for multiple pairwise comparisons, the critical *p* value for statistical significance was adjusted to *p* ≤ 0.05. Descriptive analyses will include reporting means (with SDs) for continuous variables and frequencies (with percentages) for discrete variables. One-way analysis of variance (or non-parametric Kruskal–Wallis equivalent) will be used to assess the difference in the primary outcome of CDT between those patients that received conventional therapy (without SES) and those who received SES. Differences between each patient, compared to the not affected body site, will be analyzed using paired t-test to adjust for the clustered nature of the thresholds for each patient.

## Discussion

Sensory and motor nerve function improvement following LDH, especially the influence on time and the quality of recovery, are infrequently subjected to well-designed clinical trials. There is a need for data with validated modern outcome instruments to assess evidence-based prognosis for patients with sensomotoric dysfunction. The results of this study will detect the effect of EMG-triggered multichannel SES on the improvement of mechanical and thermal sensitivity and the effect on motor recovery and pain, associated with clinical and laboratory parameters. Furthermore, additional influencing factors on sensomotoric recovery may be identified and will hopefully lead allow treatment recommendations for patients with LDH accompanied by a sensomotoric deficit.

To guarantee a high scientific value, the outcome instruments used in this trial are adjusted to the latest standards in the literature among spinal and neurologic investigations. The QST is a valuable instrument for the objectification of sensory function [25, 34]. The MMT also depicts a highly valuable tool for the quantification of muscle power and therefore we utilize the possibility to avoid an investigator’s bias, not considered in most of the previous trials. Our patient’s self-assessment (ODI, EQ5D, COMI, PD-Q, BDI) matches the highest standards of the middle European assessments for quality of life and consequently subjective efficacy of spinal procedures and management options. Patients are treated in a standardized manner and are therefore well comparable. All quantitative values can be compared between the initial treatment groups (conservative or operative) as well as between the therapy groups (SES or non-SES) and, additionally, within each patient by collecting and comparing data bilaterally (affected and non-affected side). Furthermore, our SES program was developed with leading clinical specialists and therefore matches the latest standards and innovations. Devices are especially programmed so that after the scheduled application of the medical device, individual usage times and thresholds can be read and considered by the clinical investigators.

Limitations of our trial include the expected cross-over from group conservative to group operative, as recruitment may prevail in favor of surgically treated patients. Furthermore, patients initially treated medically and randomized to the group receiving SES, will start the surgical treatment with potentially already influenced sensomotoric function. Nevertheless, before the operation a QST will be done and values will provide as reference.

Altogether, our trial was designed responsibly by experienced investigators from diverse specialties and is expected to deliver highly qualitative results in the first prospective clinical trial investigating the influence of EMG-triggered SES on the sensory and motoric recovery in patients suffering from LDH. Additionally, we expect to supply relevant outcome data on both conservative and operative management to prospectively identify a superior treatment method for symptomatic LDH.

### Trial status

Recruitment is planned to be started in October 2017, as soon as the authority’s clearance is available.

## Additional files

Additional file 1: Table S1. Primary and secondary outcome parameters. Detailed description of our primary and secondary outcome parameters. (XLS 21 kb)

Additional file 2: Table S2. Inclusion and exclusion criteria. Detailed itemization of our inclusion and exclusion criteria. (XLS 25 kb)

Additional file 3: Figure S1. Flowchart on the randomization process. Visualised radomisation allocation. (JPG 15 kb)

Additional file 4: Figure S2. Example for the placement of electrodes (here for nerve root S1 on M. gastrocnemius). (PNG 4350 kb)

## Abbreviations

ANCOVA: Analysis of covariance
DGNC: German Society of Neurosurgery
DGOOC: German Society of Orthopaedics and Orthopaedic Surgery
EMG: Electromyography
MRC: Medical Research Council
MRI: Magnetic resonance imaging
WHO: World Health Organization
