# Supplementary figures and images for: The influence of surface EMG-triggered multichannel electrical stimulation on sensomotoric recovery in patients with lumbar disc herniation: study protocol for a randomized controlled trial (RECO)
Source: Trials. 2017 Nov 25;18:566. doi: 10.1186/s13063-017-2310-z (PMC5702066; doi:10.1186/s13063-017-2310-z)

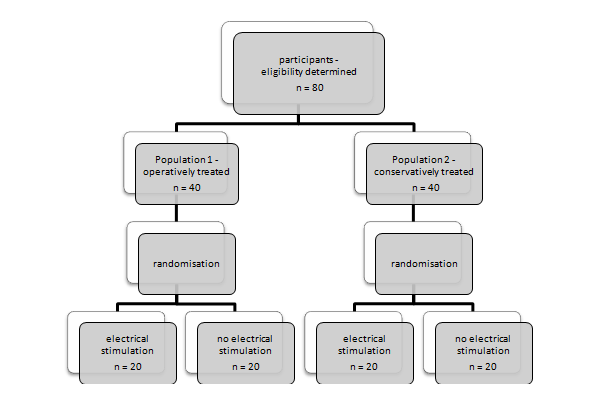

Supplement: Supplementary file 3 — Flowchart on the randomization process. Visualised radomisation allocation. (JPG 15 kb) [file 13063_2017_2310_MOESM3_ESM.jpg]

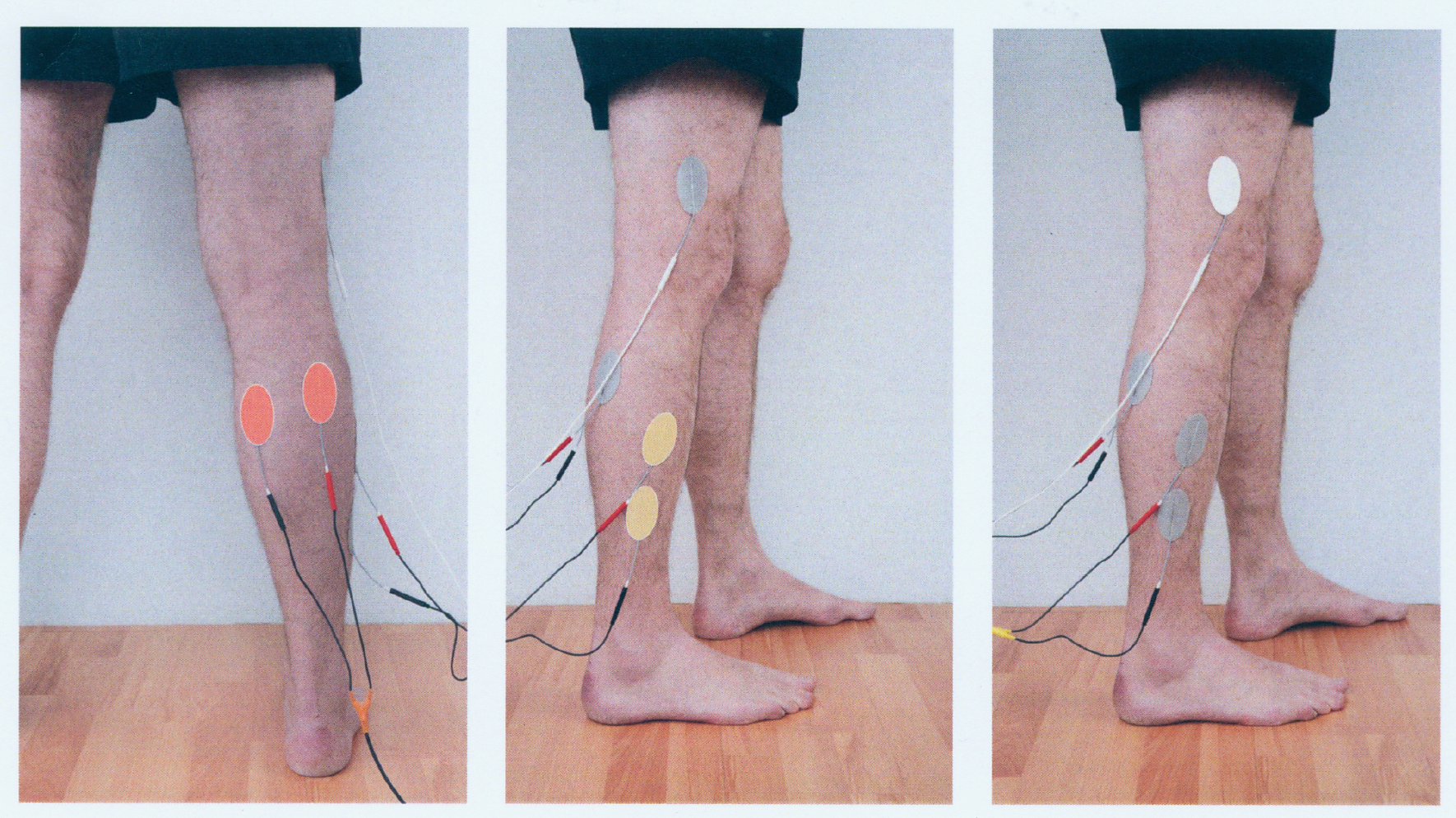

Supplement: Supplementary file 4 — Example for the placement of electrodes (here for nerve root S1 on M. gastrocnemius). (PNG 4350 kb) [file 13063_2017_2310_MOESM4_ESM.png]
